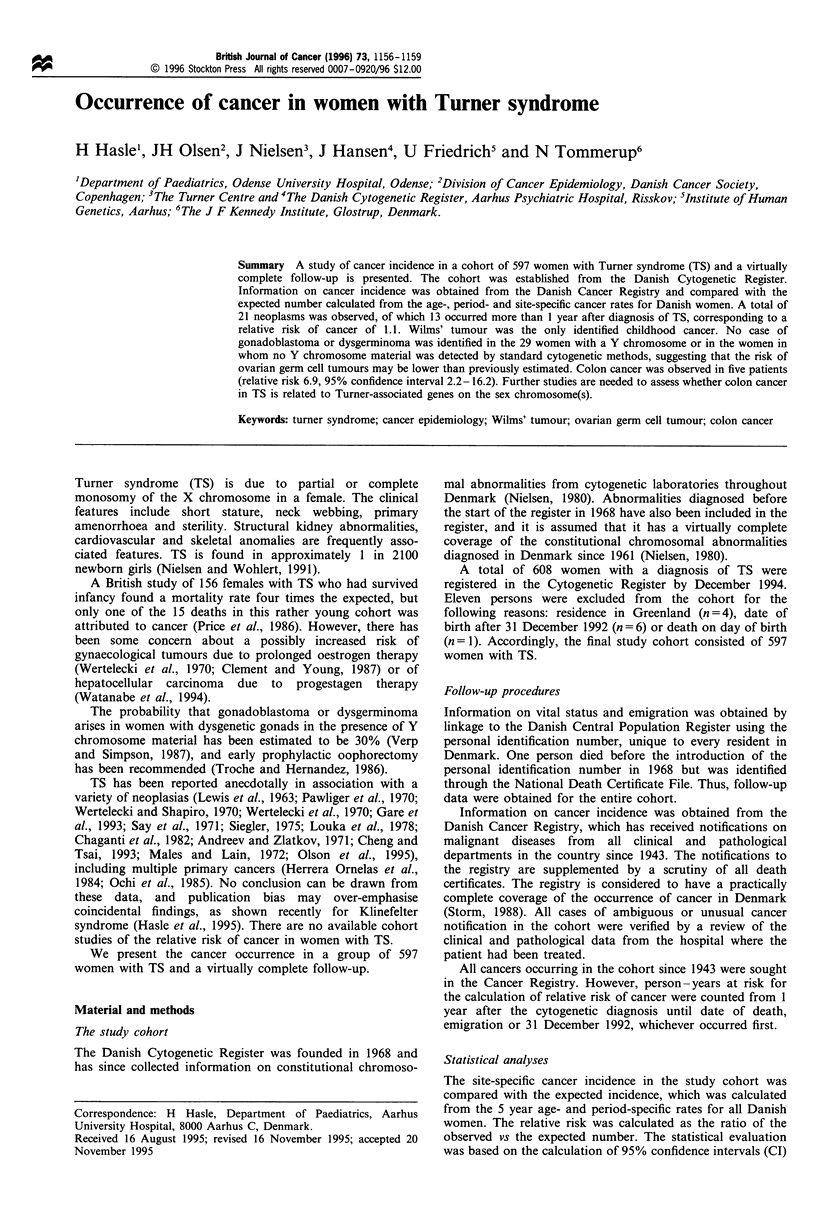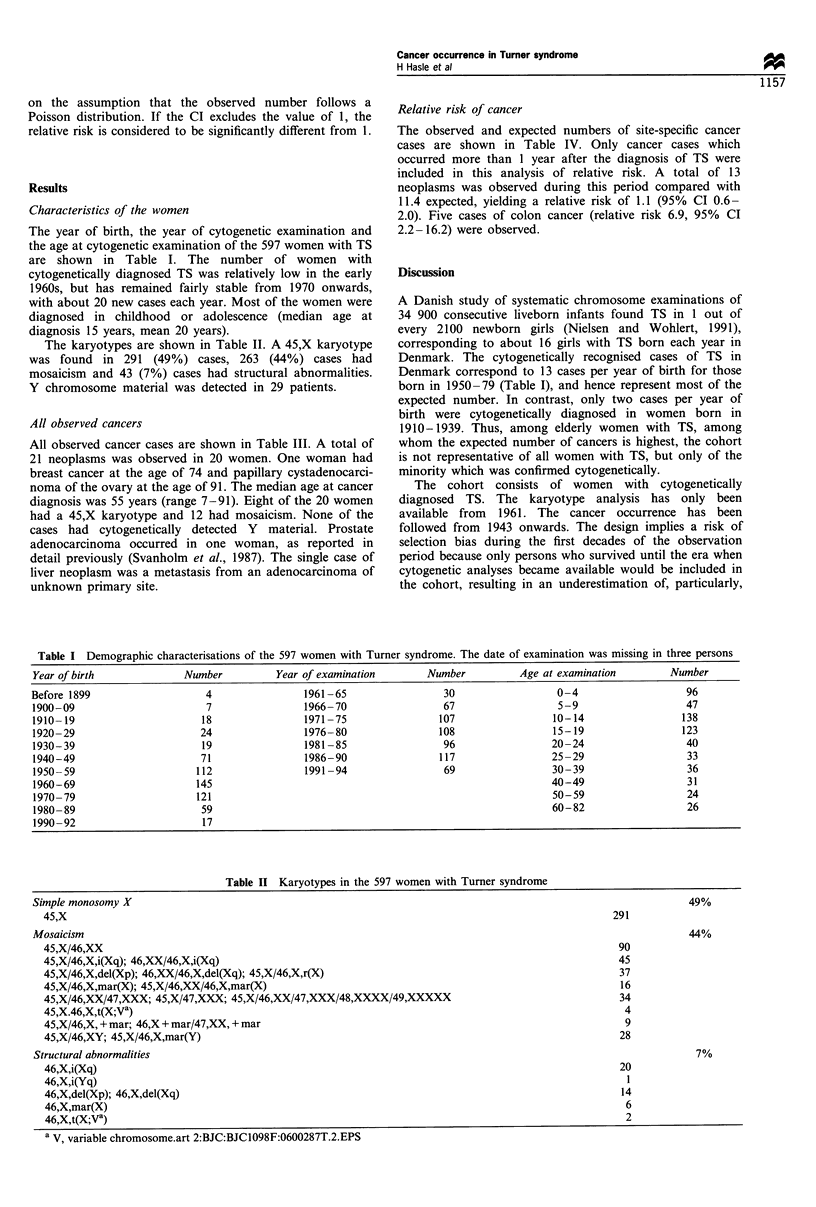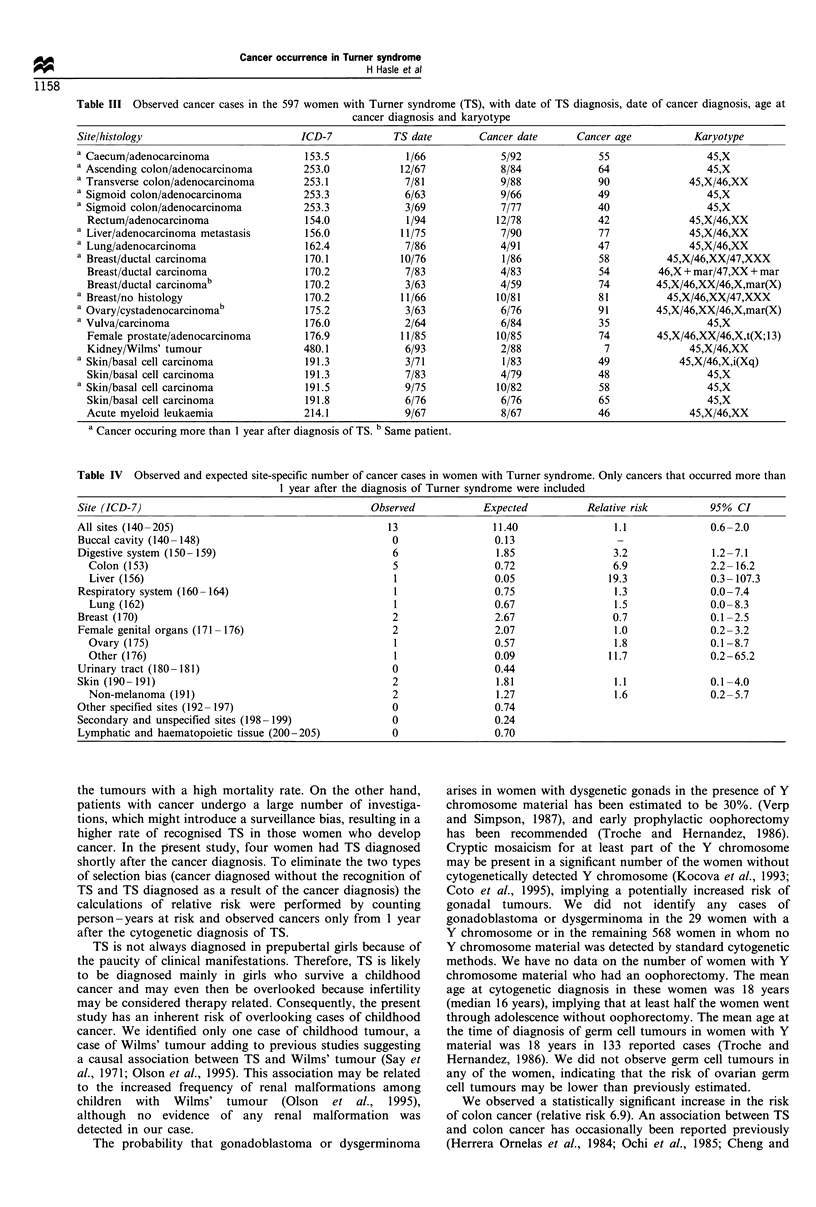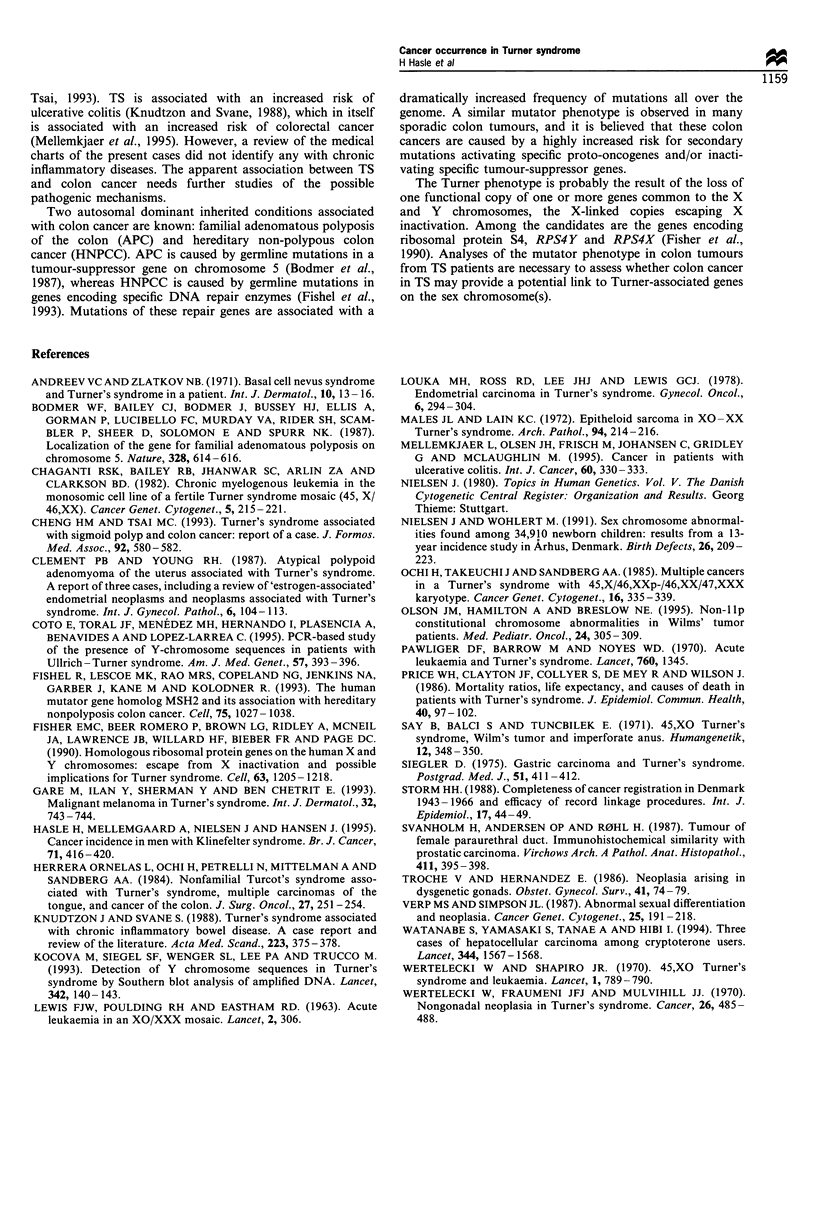# Occurrence of cancer in women with Turner syndrome.

**DOI:** 10.1038/bjc.1996.222

**Published:** 1996-05

**Authors:** H. Hasle, J. H. Olsen, J. Nielsen, J. Hansen, U. Friedrich, N. Tommerup

**Affiliations:** Department of Paediatrics, Odense University Hospital, Denmark.

## Abstract

A study of cancer incidence in a cohort of 597 women with Turner syndrome (TS) and a virtually complete follow-up is presented. The cohort was established from the Danish Cytogenetic Register. Information on cancer incidence was obtained from the Danish Cancer Registry and compared with the expected number calculated from the age-, period- and site-specific cancer rates for Danish women. A total of 21 neoplasms was observed, of which 13 occurred more than 1 year after diagnosis of TS, corresponding to a relative risk of cancer of 1.1. Wilms' tumour was the only identified childhood cancer. No case of gonadoblastoma or dysgerminoma was identified in the 29 women with a Y chromosome or in the women in whom no Y chromosome material was detected by standard cytogenetic methods, suggesting that the risk of ovarian germ cell tumours may be lower than previously estimated. Colon cancer was observed in five patients (relative risk 6.9, 95% confidence interval 2.2-16.2). Further studies are needed to assess whether colon cancer in TS is related to Turner-associated genes on the sex chromosome(s).


British Journal of Cancer (1996) 73, 1156-1159
© 1996 Stockton Press All rights reserved 0007-0920/96 $12.00

### Occurrence of cancer in women with Turner syndrome

H Hasle[1], JH Olsen[2], J Nielsen[3], J Hansen[4], U Friedrich[5] and N Tommerup[6]

[1]*Department of Paediatrics, Odense University Hospital, Odense;* [2]*Division of Cancer Epidemiology, Danish Cancer Society, Copenhagen;* [3]*The Turner Centre and* [4]*The Danish Cytogenetic Register, Aarhus Psychiatric Hospital, Risskov;* [5]*Institute of Human Genetics, Aarhus;* [6]*The J F Kennedy Institute, Glostrup, Denmark.*

**Summary** A study of cancer incidence in a cohort of 597 women with Turner syndrome (TS) and a virtually complete follow-up is presented. The cohort was established from the Danish Cytogenetic Register. Information on cancer incidence was obtained from the Danish Cancer Registry and compared with the expected number calculated from the age-, period- and site-specific cancer rates for Danish women. A total of 21 neoplasms was observed, of which 13 occurred more than 1 year after diagnosis of TS, corresponding to a relative risk of cancer of 1.1. Wilms' tumour was the only identified childhood cancer. No case of gonadoblastoma or dysgerminoma was identified in the 29 women with a Y chromosome or in the women in whom no Y chromosome material was detected by standard cytogenetic methods, suggesting that the risk of ovarian germ cell tumours may be lower than previously estimated. Colon cancer was observed in five patients (relative risk 6.9, 95% confidence interval 2.2-16.2). Further studies are needed to assess whether colon cancer in TS is related to Turner-associated genes on the sex chromosome(s).

**Keywords:** turner syndrome; cancer epidemiology; Wilms' tumour; ovarian germ cell tumour; colon cancer

Turner syndrome (TS) is due to partial or complete monosomy of the X chromosome in a female. The clinical features include short stature, neck webbing, primary amenorrhoea and sterility. Structural kidney abnormalities, cardiovascular and skeletal anomalies are frequently associated features. TS is found in approximately 1 in 2100 newborn girls (Nielsen and Wohlert, 1991).

A British study of 156 females with TS who had survived infancy found a mortality rate four times the expected, but only one of the 15 deaths in this rather young cohort was attributed to cancer (Price *et al.*, 1986). However, there has been some concern about a possibly increased risk of gynaecological tumours due to prolonged oestrogen therapy (Wertelecki *et al.*, 1970; Clement and Young, 1987) or of hepatocellular carcinoma due to progestagen therapy (Watanabe *et al.*, 1994).

The probability that gonadoblastoma or dysgerminoma arises in women with dysgenetic gonads in the presence of Y chromosome material has been estimated to be 30% (Verp and Simpson, 1987), and early prophylactic oophorectomy has been recommended (Troche and Hernandez, 1986).

TS has been reported anecdotally in association with a variety of neoplasias (Lewis *et al.*, 1963; Pawliger *et al.*, 1970; Wertelecki and Shapiro, 1970; Wertelecki *et al.*, 1970; Gare *et al.*, 1993; Say *et al.*, 1971; Siegler, 1975; Louka *et al.*, 1978; Chaganti *et al.*, 1982; Andreev and Zlatkov, 1971; Cheng and Tsai, 1993; Males and Lain, 1972; Olson *et al.*, 1995), including multiple primary cancers (Herrera Ornelas *et al.*, 1984; Ochi *et al.*, 1985). No conclusion can be drawn from these data, and publication bias may over-emphasise coincidental findings, as shown recently for Klinefelter syndrome (Hasle *et al.*, 1995). There are no available cohort studies of the relative risk of cancer in women with TS.

We present the cancer occurrence in a group of 597 women with TS and a virtually complete follow-up.

### Material and methods

### *The study cohort*

The Danish Cytogenetic Register was founded in 1968 and has since collected information on constitutional chromosomal abnormalities from cytogenetic laboratories throughout Denmark (Nielsen, 1980). Abnormalities diagnosed before the start of the register in 1968 have also been included in the register, and it is assumed that it has a virtually complete coverage of the constitutional chromosomal abnormalities diagnosed in Denmark since 1961 (Nielsen, 1980).

A total of 608 women with a diagnosis of TS were registered in the Cytogenetic Register by December 1994. Eleven persons were excluded from the cohort for the following reasons: residence in Greenland ($n=4$), date of birth after 31 December 1992 ($n=6$) or death on day of birth ($n=1$). Accordingly, the final study cohort consisted of 597 women with TS.

### *Follow-up procedures*

Information on vital status and emigration was obtained by linkage to the Danish Central Population Register using the personal identification number, unique to every resident in Denmark. One person died before the introduction of the personal identification number in 1968 but was identified through the National Death Certificate File. Thus, follow-up data were obtained for the entire cohort.

Information on cancer incidence was obtained from the Danish Cancer Registry, which has received notifications on malignant diseases from all clinical and pathological departments in the country since 1943. The notifications to the registry are supplemented by a scrutiny of all death certificates. The registry is considered to have a practically complete coverage of the occurrence of cancer in Denmark (Storm, 1988). All cases of ambiguous or unusual cancer notification in the cohort were verified by a review of the clinical and pathological data from the hospital where the patient had been treated.

All cancers occurring in the cohort since 1943 were sought in the Cancer Registry. However, person-years at risk for the calculation of relative risk of cancer were counted from 1 year after the cytogenetic diagnosis until date of death, emigration or 31 December 1992, whichever occurred first.

### *Statistical analyses*

The site-specific cancer incidence in the study cohort was compared with the expected incidence, which was calculated from the 5 year age- and period-specific rates for all Danish women. The relative risk was calculated as the ratio of the observed *vs* the expected number. The statistical evaluation was based on the calculation of 95% confidence intervals (CI)

Correspondence: H Hasle, Department of Paediatrics, Aarhus University Hospital, 8000 Aarhus C, Denmark.
Received 16 August 1995; revised 16 November 1995; accepted 20 November 1995

on the assumption that the observed number follows a Poisson distribution. If the CI excludes the value of 1, the relative risk is considered to be significantly different from 1.

### Results

*Characteristics of the women*

The year of birth, the year of cytogenetic examination and the age at cytogenetic examination of the 597 women with TS are shown in Table I. The number of women with cytogenetically diagnosed TS was relatively low in the early 1960s, but has remained fairly stable from 1970 onwards, with about 20 new cases each year. Most of the women were diagnosed in childhood or adolescence (median age at diagnosis 15 years, mean 20 years).

The karyotypes are shown in Table II. A 45,X karyotype was found in 291 (49%) cases, 263 (44%) cases had mosaicism and 43 (7%) cases had structural abnormalities. Y chromosome material was detected in 29 patients.

*All observed cancers*

All observed cancer cases are shown in Table III. A total of 21 neoplasms was observed in 20 women. One woman had breast cancer at the age of 74 and papillary cystadenocarcinoma of the ovary at the age of 91. The median age at cancer diagnosis was 55 years (range 7–91). Eight of the 20 women had a 45,X karyotype and 12 had mosaicism. None of the cases had cytogenetically detected Y material. Prostate adenocarcinoma occurred in one woman, as reported in detail previously (Svanholm *et al.*, 1987). The single case of liver neoplasm was a metastasis from an adenocarcinoma of unknown primary site.

*Relative risk of cancer*

The observed and expected numbers of site-specific cancer cases are shown in Table IV. Only cancer cases which occurred more than 1 year after the diagnosis of TS were included in this analysis of relative risk. A total of 13 neoplasms was observed during this period compared with 11.4 expected, yielding a relative risk of 1.1 (95% CI 0.6–2.0). Five cases of colon cancer (relative risk 6.9, 95% CI 2.2–16.2) were observed.

### Discussion

A Danish study of systematic chromosome examinations of 34 900 consecutive liveborn infants found TS in 1 out of every 2100 newborn girls (Nielsen and Wohlert, 1991), corresponding to about 16 girls with TS born each year in Denmark. The cytogenetically recognised cases of TS in Denmark correspond to 13 cases per year of birth for those born in 1950–79 (Table I), and hence represent most of the expected number. In contrast, only two cases per year of birth were cytogenetically diagnosed in women born in 1910–1939. Thus, among elderly women with TS, among whom the expected number of cancers is highest, the cohort is not representative of all women with TS, but only of the minority which was confirmed cytogenetically.

The cohort consists of women with cytogenetically diagnosed TS. The karyotype analysis has only been available from 1961. The cancer occurrence has been followed from 1943 onwards. The design implies a risk of selection bias during the first decades of the observation period because only persons who survived until the era when cytogenetic analyses became available would be included in the cohort, resulting in an underestimation of, particularly,

**Table I** Demographic characterisations of the 597 women with Turner syndrome. The date of examination was missing in three persons

| *Year of birth* | *Number* | *Year of examination* | *Number* | *Age at examination* | *Number* |
|---|---|---|---|---|---|
| Before 1899 | 4 | 1961–65 | 30 | 0–4 | 96 |
| 1900–09 | 7 | 1966–70 | 67 | 5–9 | 47 |
| 1910–19 | 18 | 1971–75 | 107 | 10–14 | 138 |
| 1920–29 | 24 | 1976–80 | 108 | 15–19 | 123 |
| 1930–39 | 19 | 1981–85 | 96 | 20–24 | 40 |
| 1940–49 | 71 | 1986–90 | 117 | 25–29 | 33 |
| 1950–59 | 112 | 1991–94 | 69 | 30–39 | 36 |
| 1960–69 | 145 | | | 40–49 | 31 |
| 1970–79 | 121 | | | 50–59 | 24 |
| 1980–89 | 59 | | | 60–82 | 26 |
| 1990–92 | 17 | | | | |

**Table II** Karyotypes in the 597 women with Turner syndrome

| | | |
|---|---|---|
| *Simple monosomy X* | | 49% |
| 45,X | 291 | |
| *Mosaicism* | | 44% |
| 45,X/46,XX | 90 | |
| 45,X/46,X,i(Xq); 46,XX/46,X,i(Xq) | 45 | |
| 45,X/46,X,del(Xp); 46,XX/46,X,del(Xq); 45,X/46,X,r(X) | 37 | |
| 45,X/46,X,mar(X); 45,X/46,XX/46,X,mar(X) | 16 | |
| 45,X/46,XX/47,XXX; 45,X/47,XXX; 45,X/46,XX/47,XXX/48,XXXX/49,XXXXX | 34 | |
| 45,X.46,X,t(X;V[a]) | 4 | |
| 45,X/46,X,+mar; 46,X+mar/47,XX,+mar | 9 | |
| 45,X/46,XY; 45,X/46,X,mar(Y) | 28 | |
| *Structural abnormalities* | | 7% |
| 46,X,i(Xq) | 20 | |
| 46,X,i(Yq) | 1 | |
| 46,X,del(Xp); 46,X,del(Xq) | 14 | |
| 46,X,mar(X) | 6 | |
| 46,X,t(X;V[a]) | 2 | |

[a] V, variable chromosome.art 2:BJC:BJC1098F:0600287T.2.EPS

Table III Observed cancer cases in the 597 women with Turner syndrome (TS), with date of TS diagnosis, date of cancer diagnosis, age at cancer diagnosis and karyotype

| Site/histology | ICD-7 | TS date | Cancer date | Cancer age | Karyotype |
|---|---|---|---|---|---|
| [a] Caecum/adenocarcinoma | 153.5 | 1/66 | 5/92 | 55 | 45,X |
| [a] Ascending colon/adenocarcinoma | 253.0 | 12/67 | 8/84 | 64 | 45,X |
| [a] Transverse colon/adenocarcinoma | 253.1 | 7/81 | 9/88 | 90 | 45,X/46,XX |
| [a] Sigmoid colon/adenocarcinoma | 253.3 | 6/63 | 9/66 | 49 | 45,X |
| [a] Sigmoid colon/adenocarcinoma | 253.3 | 3/69 | 7/77 | 40 | 45,X |
| Rectum/adenocarcinoma | 154.0 | 1/94 | 12/78 | 42 | 45,X/46,XX |
| [a] Liver/adenocarcinoma metastasis | 156.0 | 11/75 | 7/90 | 77 | 45,X/46,XX |
| [a] Lung/adenocarcinoma | 162.4 | 7/86 | 4/91 | 47 | 45,X/46,XX |
| [a] Breast/ductal carcinoma | 170.1 | 10/76 | 1/86 | 58 | 45,X/46,XX/47,XXX |
| Breast/ductal carcinoma | 170.2 | 7/83 | 4/83 | 54 | 46,X + mar/47,XX + mar |
| Breast/ductal carcinoma[b] | 170.2 | 3/63 | 4/59 | 74 | 45,X/46,XX/46,X,mar(X) |
| [a] Breast/no histology | 170.2 | 11/66 | 10/81 | 81 | 45,X/46,XX/47,XXX |
| [a] Ovary/cystadenocarcinoma[b] | 175.2 | 3/63 | 6/76 | 91 | 45,X/46,XX/46,X,mar(X) |
| [a] Vulva/carcinoma | 176.0 | 2/64 | 6/84 | 35 | 45,X |
| Female prostate/adenocarcinoma | 176.9 | 11/85 | 10/85 | 74 | 45,X/46,XX/46,X,t(X;13) |
| Kidney/Wilms' tumour | 480.1 | 6/93 | 2/88 | 7 | 45,X/46,XX |
| [a] Skin/basal cell carcinoma | 191.3 | 3/71 | 1/83 | 49 | 45,X/46,X,i(Xq) |
| Skin/basal cell carcinoma | 191.3 | 7/83 | 4/79 | 48 | 45,X |
| [a] Skin/basal cell carcinoma | 191.5 | 9/75 | 10/82 | 58 | 45,X |
| Skin/basal cell carcinoma | 191.8 | 6/76 | 6/76 | 65 | 45,X |
| Acute myeloid leukaemia | 214.1 | 9/67 | 8/67 | 46 | 45,X/46,XX |

[a] Cancer occuring more than 1 year after diagnosis of TS. [b] Same patient.

Table IV Observed and expected site-specific number of cancer cases in women with Turner syndrome. Only cancers that occurred more than 1 year after the diagnosis of Turner syndrome were included

| Site (ICD-7) | Observed | Expected | Relative risk | 95% CI |
|---|---|---|---|---|
| All sites (140–205) | 13 | 11.40 | 1.1 | 0.6–2.0 |
| Buccal cavity (140–148) | 0 | 0.13 | – | |
| Digestive system (150–159) | 6 | 1.85 | 3.2 | 1.2–7.1 |
| Colon (153) | 5 | 0.72 | 6.9 | 2.2–16.2 |
| Liver (156) | 1 | 0.05 | 19.3 | 0.3–107.3 |
| Respiratory system (160–164) | 1 | 0.75 | 1.3 | 0.0–7.4 |
| Lung (162) | 1 | 0.67 | 1.5 | 0.0–8.3 |
| Breast (170) | 2 | 2.67 | 0.7 | 0.1–2.5 |
| Female genital organs (171–176) | 2 | 2.07 | 1.0 | 0.2–3.2 |
| Ovary (175) | 1 | 0.57 | 1.8 | 0.1–8.7 |
| Other (176) | 1 | 0.09 | 11.7 | 0.2–65.2 |
| Urinary tract (180–181) | 0 | 0.44 | | |
| Skin (190–191) | 2 | 1.81 | 1.1 | 0.1–4.0 |
| Non-melanoma (191) | 2 | 1.27 | 1.6 | 0.2–5.7 |
| Other specified sites (192–197) | 0 | 0.74 | | |
| Secondary and unspecified sites (198–199) | 0 | 0.24 | | |
| Lymphatic and haematopoietic tissue (200–205) | 0 | 0.70 | | |

the tumours with a high mortality rate. On the other hand, patients with cancer undergo a large number of investigations, which might introduce a surveillance bias, resulting in a higher rate of recognised TS in those women who develop cancer. In the present study, four women had TS diagnosed shortly after the cancer diagnosis. To eliminate the two types of selection bias (cancer diagnosed without the recognition of TS and TS diagnosed as a result of the cancer diagnosis) the calculations of relative risk were performed by counting person–years at risk and observed cancers only from 1 year after the cytogenetic diagnosis of TS.

TS is not always diagnosed in prepubertal girls because of the paucity of clinical manifestations. Therefore, TS is likely to be diagnosed mainly in girls who survive a childhood cancer and may even then be overlooked because infertility may be considered therapy related. Consequently, the present study has an inherent risk of overlooking cases of childhood cancer. We identified only one case of childhood tumour, a case of Wilms' tumour adding to previous studies suggesting a causal association between TS and Wilms' tumour (Say *et al.*, 1971; Olson *et al.*, 1995). This association may be related to the increased frequency of renal malformations among children with Wilms' tumour (Olson *et al.*, 1995), although no evidence of any renal malformation was detected in our case.

The probability that gonadoblastoma or dysgerminoma arises in women with dysgenetic gonads in the presence of Y chromosome material has been estimated to be 30%. (Verp and Simpson, 1987), and early prophylactic oophorectomy has been recommended (Troche and Hernandez, 1986). Cryptic mosaicism for at least part of the Y chromosome may be present in a significant number of the women without cytogenetically detected Y chromosome (Kocova *et al.*, 1993; Coto *et al.*, 1995), implying a potentially increased risk of gonadal tumours. We did not identify any cases of gonadoblastoma or dysgerminoma in the 29 women with a Y chromosome or in the remaining 568 women in whom no Y chromosome material was detected by standard cytogenetic methods. We have no data on the number of women with Y chromosome material who had an oophorectomy. The mean age at cytogenetic diagnosis in these women was 18 years (median 16 years), implying that at least half the women went through adolescence without oophorectomy. The mean age at the time of diagnosis of germ cell tumours in women with Y material was 18 years in 133 reported cases (Troche and Hernandez, 1986). We did not observe germ cell tumours in any of the women, indicating that the risk of ovarian germ cell tumours may be lower than previously estimated.

We observed a statistically significant increase in the risk of colon cancer (relative risk 6.9). An association between TS and colon cancer has occasionally been reported previously (Herrera Ornelas *et al.*, 1984; Ochi *et al.*, 1985; Cheng and

Tsai, 1993). TS is associated with an increased risk of ulcerative colitis (Knudtzon and Svane, 1988), which in itself is associated with an increased risk of colorectal cancer (Mellemkjaer *et al.*, 1995). However, a review of the medical charts of the present cases did not identify any with chronic inflammatory diseases. The apparent association between TS and colon cancer needs further studies of the possible pathogenic mechanisms.

Two autosomal dominant inherited conditions associated with colon cancer are known: familial adenomatous polyposis of the colon (APC) and hereditary non-polypous colon cancer (HNPCC). APC is caused by germline mutations in a tumour-suppressor gene on chromosome 5 (Bodmer *et al.*, 1987), whereas HNPCC is caused by germline mutations in genes encoding specific DNA repair enzymes (Fishel *et al.*, 1993). Mutations of these repair genes are associated with a dramatically increased frequency of mutations all over the genome. A similar mutator phenotype is observed in many sporadic colon tumours, and it is believed that these colon cancers are caused by a highly increased risk for secondary mutations activating specific proto-oncogenes and/or inactivating specific tumour-suppressor genes.

The Turner phenotype is probably the result of the loss of one functional copy of one or more genes common to the X and Y chromosomes, the X-linked copies escaping X inactivation. Among the candidates are the genes encoding ribosomal protein S4, *RPS4Y* and *RPS4X* (Fisher *et al.*, 1990). Analyses of the mutator phenotype in colon tumours from TS patients are necessary to assess whether colon cancer in TS may provide a potential link to Turner-associated genes on the sex chromosome(s).

### References

ANDREEV VC AND ZLATKOV NB. (1971). Basal cell nevus syndrome and Turner's syndrome in a patient. *Int. J. Dermatol.*, **10**, 13-16.

BODMER WF, BAILEY CJ, BODMER J, BUSSEY HJ, ELLIS A, GORMAN P, LUCIBELLO FC, MURDAY VA, RIDER SH, SCAMBLER P, SHEER D, SOLOMON E AND SPURR NK. (1987). Localization of the gene for familial adenomatous polyposis on chromosome 5. *Nature*, **328**, 614-616.

CHAGANTI RSK, BAILEY RB, JHANWAR SC, ARLIN ZA AND CLARKSON BD. (1982). Chronic myelogenous leukemia in the monosomic cell line of a fertile Turner syndrome mosaic (45, X/46,XX). *Cancer Genet. Cytogenet.*, **5**, 215-221.

CHENG HM AND TSAI MC. (1993). Turner's syndrome associated with sigmoid polyp and colon cancer: report of a case. *J. Formos. Med. Assoc.*, **92**, 580-582.

CLEMENT PB AND YOUNG RH. (1987). Atypical polypoid adenomyoma of the uterus associated with Turner's syndrome. A report of three cases, including a review of 'estrogen-associated' endometrial neoplasms and neoplasms associated with Turner's syndrome. *Int. J. Gynecol. Pathol.*, **6**, 104-113.

COTO E, TORAL JF, MENÉDEZ MH, HERNANDO I, PLASENCIA A, BENAVIDES A AND LOPEZ-LARREA C. (1995). PCR-based study of the presence of Y-chromosome sequences in patients with Ullrich-Turner syndrome. *Am. J. Med. Genet.*, **57**, 393-396.

FISHEL R, LESCOE MK, RAO MRS, COPELAND NG, JENKINS NA, GARBER J, KANE M AND KOLODNER R. (1993). The human mutator gene homolog MSH2 and its association with hereditary nonpolyposis colon cancer. *Cell*, **75**, 1027-1038.

FISHER EMC, BEER ROMERO P, BROWN LG, RIDLEY A, MCNEIL JA, LAWRENCE JB, WILLARD HF, BIEBER FR AND PAGE DC. (1990). Homologous ribosomal protein genes on the human X and Y chromosomes: escape from X inactivation and possible implications for Turner syndrome. *Cell*, **63**, 1205-1218.

GARE M, ILAN Y, SHERMAN Y AND BEN CHETRIT E. (1993). Malignant melanoma in Turner's syndrome. *Int. J. Dermatol.*, **32**, 743-744.

HASLE H, MELLEMGAARD A, NIELSEN J AND HANSEN J. (1995). Cancer incidence in men with Klinefelter syndrome. *Br. J. Cancer*, **71**, 416-420.

HERRERA ORNELAS L, OCHI H, PETRELLI N, MITTELMAN A AND SANDBERG AA. (1984). Nonfamilial Turcot's syndrome associated with Turner's syndrome, multiple carcinomas of the tongue, and cancer of the colon. *J. Surg. Oncol.*, **27**, 251-254.

KNUDTZON J AND SVANE S. (1988). Turner's syndrome associated with chronic inflammatory bowel disease. A case report and review of the literature. *Acta Med. Scand.*, **223**, 375-378.

KOCOVA M, SIEGEL SF, WENGER SL, LEE PA AND TRUCCO M. (1993). Detection of Y chromosome sequences in Turner's syndrome by Southern blot analysis of amplified DNA. *Lancet*, **342**, 140-143.

LEWIS FJW, POULDING RH AND EASTHAM RD. (1963). Acute leukaemia in an XO/XXX mosaic. *Lancet*, **2**, 306.

LOUKA MH, ROSS RD, LEE JHJ AND LEWIS GCJ. (1978). Endometrial carcinoma in Turner's syndrome. *Gynecol. Oncol.*, **6**, 294-304.

MALES JL AND LAIN KC. (1972). Epitheloid sarcoma in XO-XX Turner's syndrome. *Arch. Pathol.*, **94**, 214-216.

MELLEMKJAER L, OLSEN JH, FRISCH M, JOHANSEN C, GRIDLEY G AND MCLAUGHLIN M. (1995). Cancer in patients with ulcerative colitis. *Int. J. Cancer*, **60**, 330-333.

NIELSEN J. (1980). *Topics in Human Genetics. Vol. V. The Danish Cytogenetic Central Register: Organization and Results.* Georg Thieme: Stuttgart.

NIELSEN J AND WOHLERT M. (1991). Sex chromosome abnormalities found among 34,910 newborn children: results from a 13-year incidence study in Århus, Denmark. *Birth Defects*, **26**, 209-223.

OCHI H, TAKEUCHI J AND SANDBERG AA. (1985). Multiple cancers in a Turner's syndrome with 45,X/46,XXp-/46,XX/47,XXX karyotype. *Cancer Genet. Cytogenet.*, **16**, 335-339.

OLSON JM, HAMILTON A AND BRESLOW NE. (1995). Non-11p constitutional chromosome abnormalities in Wilms' tumor patients. *Med. Pediatr. Oncol.*, **24**, 305-309.

PAWLIGER DF, BARROW M AND NOYES WD. (1970). Acute leukaemia and Turner's syndrome. *Lancet*, **760**, 1345.

PRICE WH, CLAYTON JF, COLLYER S, DE MEY R AND WILSON J. (1986). Mortality ratios, life expectancy, and causes of death in patients with Turner's syndrome. *J. Epidemiol. Commun. Health*, **40**, 97-102.

SAY B, BALCI S AND TUNCBILEK E. (1971). 45,XO Turner's syndrome, Wilm's tumor and imperforate anus. *Humangenetik*, **12**, 348-350.

SIEGLER D. (1975). Gastric carcinoma and Turner's syndrome. *Postgrad. Med. J.*, **51**, 411-412.

STORM HH. (1988). Completeness of cancer registration in Denmark 1943-1966 and efficacy of record linkage procedures. *Int. J. Epidemiol.*, **17**, 44-49.

SVANHOLM H, ANDERSEN OP AND RØHL H. (1987). Tumour of female paraurethral duct. Immunohistochemical similarity with prostatic carcinoma. *Virchows Arch. A Pathol. Anat. Histopathol.*, **411**, 395-398.

TROCHE V AND HERNANDEZ E. (1986). Neoplasia arising in dysgenetic gonads. *Obstet. Gynecol. Surv.*, **41**, 74-79.

VERP MS AND SIMPSON JL. (1987). Abnormal sexual differentiation and neoplasia. *Cancer Genet. Cytogenet.*, **25**, 191-218.

WATANABE S, YAMASAKI S, TANAE A AND HIBI I. (1994). Three cases of hepatocellular carcinoma among cryptoterone users. *Lancet*, **344**, 1567-1568.

WERTELECKI W AND SHAPIRO JR. (1970). 45,XO Turner's syndrome and leukaemia. *Lancet*, **1**, 789-790.

WERTELECKI W, FRAUMENI JFJ AND MULVIHILL JJ. (1970). Nongonadal neoplasia in Turner's syndrome. *Cancer*, **26**, 485-488.